# Society Meetings

**Published:** 1903-02

**Authors:** 


### SOCIETY MEETINGS.

---

The Medical Society of the County of Erie held its 82d annual meeting, January 13, 1903, at which the following-named officers were elected: President, Ernest Wende, Buffalo; vice-president, J. D. Macpherson, Akron; secretary, F. C. Gram, Buffalo; treasurer, Edward Clark, Buffalo; librarian, W. C. Callinan, Buffalo; censors, Irving W. Potter, John B. Coakley, Henry R. Hopkins, Henry Lapp and F. E. Fronczak.

---

The Wyoming County Medical Association held its semiannual meeting at Perry, January 6, 1903, with the following program: Syphilis of the nervous system, Floyd S. Crego, Buffalo; Eczema, Grover W. Wende, Buffalo; Two centuries of smallpox, the one before and the one after the discovery of vaccination, Z. J. Lusk, Warsaw; Injuries to the shoulder joint, E. B. Belknap, Wyoming.

---

The Buffalo Academy of Medicine held meetings during the months of December and January, 1902 and 1903, as follows:

*Section on Ophthalmology, Otology and Laryngology.*—Tuesday evening, December 30. Program: Some remarks on nasal and pharyngeal reflex neuroses, W. S. Renner. On the present status of the vaccine question, as shown by inoculation experiments upon the cornea, H. R. Gaylord.

*Section on Surgery.*—Tuesday evening, January 6. Program: Some of the causes leading to and producing suppuration, F. W. McGuire. Operative treatment of clubfoot, Bernard Bartow.

*Section on Medicine.*—Tuesday evening, January 13. Program: Infantile eclampsia, A. J. Colton. Injuries and infections of newly-born children, Irving M. Snow.

*Section on Pathology.*—Tuesday evening, January 20. Program: Universal diseases seen in Buffalo, imported from our tropical possessions, with demonstrations by patients from Fort Porter, John J. Riley, U.S.A., Surgeon-major at Fort Porter.

